# Williams syndrome and psychosis: a case report

**DOI:** 10.1186/1752-1947-8-49

**Published:** 2014-02-12

**Authors:** Henrique Salgado, Luís Martins-Correia

**Affiliations:** 1 Hospital de Magalhães Lemos, Rua Professor Álvaro Rodrigues, Porto, 4149-003, Portugal

**Keywords:** Developmental disability, Intellectual disability, Psychosis, Williams syndrome

## Abstract

**Introduction:**

Mental comorbidities, such as phobia, obsessive compulsive symptoms and anxiety disorders, are common in Williams syndrome. However, psychotic symptoms are rare in these patients. We report a case of psychosis in a patient with Williams syndrome.

**Case presentation:**

A 23-year-old Caucasian woman with Williams syndrome arrived at the emergency room with persecutory delusions, auditory and verbal hallucinations, soliloquies and psychomotor agitation. These symptoms were consistently present for 2 months. No evidence of other medical illnesses or psychoactive substances was found. There was no evidence of prior psychiatric symptoms or family history of psychiatric or neurological disorders. She was treated with antipsychotics and her symptoms were resolved.

**Conclusion:**

We describe a rare case of a patient with Williams syndrome who experienced a nonorganic psychotic episode. Literature on this topic is scarce and, therefore, this case report intends to add further data about this comorbidity.

## Introduction

Williams syndrome (WS) is a neurodevelopmental disorder caused by a deletion of genes on chromosome 7q11.23 [1,2] with a potential impact on virtually all organ systems [2]. Clinical suspicion is essential because the diagnosis is set through genetic studies, which are not performed by routine chromosomal analysis [3].

The WS phenotype is variable [4], but it is associated with a distinctive pattern of cognitive abilities [5]. In particular, these patients show a severe visuospatial construction deficit in contrast with a relative strength in verbal short-term memory, language [1] and unusual musical talent [1]. Furthermore, clinical findings include distinct facies (elf-like face [6]), cardiovascular abnormalities, growth retardation, connective tissue abnormalities and endocrine abnormalities, such as hypercalcemia, hypercalciuria and early, although not precocious, puberty [4,7].

This syndrome could account for 6% of all cases of mental retardation of genetic origin [1]. Also, there is increasing evidence that many syndromes involving mental retardation are associated with not only specific medical and cognitive phenotypes but also particular patterns of behavioral and psychiatric traits [8]. Individuals with WS often have a personality and behavior pattern that includes distractibility, restlessness, social disinhibition, excessive talking, mood swings and anxiety [8], the latter of which is one of the most distinguishable features of this behavioral phenotype [5]. Anxiety is the most common psychiatric comorbidity among these patients, with up to 54% meeting International Classification of Diseases/*Diagnostic and Statistical Manual of Mental Disorders* diagnostic criteria for anxiety disorders (for example agoraphobia, generalized anxiety disorder and specific phobia). However, other psychiatric disorders are much less common in WS [5]. Psychotic symptoms in these patients are rare and descriptions in the literature of such comorbidity are very scarce. Here, the authors present a case of a patient with WS who developed an acute psychotic episode.

## Case presentation

A 23-year-old Caucasian woman who suffers from WS was evaluated in the emergency room after being injured in a fall. She had left her home during the night without being noticed by her family. She fell next to a highway near her house and fractured her leg; a driver found her, took her to the nearest hospital and contacted her relatives. She was evaluated by orthopedics and then sent home with her family with orders to be medicated with acetaminophen 1000mg three times a day (t.i.d.) alternated with ibuprofen 600mg t.i.d. At home, she presented with psychomotor agitation; her family decided to take her back to the hospital only a few hours after she was discharged. She was presenting with persecutory delusions, auditory and verbal hallucinations, soliloquies, and psychic and motor agitation, and was hospitalized.

Apart from the WS, she had no known medical illness and was not taking any medication before being admitted to the hospital. Nevertheless, she was submitted to extensive medical investigation in order to exclude possible organic causes for the symptoms. A cerebral computed tomography scan did not reveal any lesions. Abdominal ultrasonography showed a slight increase of diffuse liver echogenicity, with no other changes. Her total and direct bilirubin levels were higher than normal (1.8mg/dL and 0.6mg/dL, respectively). No abnormalities were found in her renal function (creatinine, urea and glomerular filtration rate), electrolytes, liver function tests and C-reactive protein. A full blood count showed normal values, as did thyroid-stimulating hormone, free thyroxine and haptoglobin. Viral markers for human immunodeficiency virus 1 and 2, hepatitis C virus and hepatitis C virus were negative. Her toxicology screening was negative as well. Meanwhile, haloperidol 6mg daily was introduced. Liaison psychiatry later confirmed the presence of these symptoms and haloperidol was therefore maintained. As no acute organic cause for her symptoms was found, she was transferred to our psychiatric unit 2 days after being hospitalized. A mental examination was difficult to execute due to the patient’s intellectual disability, but family members provided considerable valuable information. She was still presenting with persecutory delusions and auditory-verbal hallucinations at this time.

On the third day of receiving haloperidol 6mg daily, she developed medication-specific side effects: sialorrhea, excessive somnolence and cogwheel rigidity, primarily on her upper left limb. The haloperidol was replaced with risperidone 2mg daily. The side effects improved, but as she was still presenting with excessive somnolence, the risperidone dosage was decreased to 1.5mg daily.

Approximately 1 year before this episode, she had been expelled from a geriatric care unit after she began to exhibit inappropriate behavior. Her family also reported that she quite frequently had tried to leave home until 2 months before being hospitalized, when her behavior changed. She began to be more suspicious and afraid of being alone. She would look behind doors and to the ceiling as if she expected to find something there. Her mother said in an interview, “She spoke very often to herself, but in a manner that looked like there was someone else there with her.” Her mother associated these changes in her behavior with specific events. Namely, she said this behavior began when the patient’s younger brother (who had looked after her during the past year) resumed school and when another brother and her stepfather fought, resulting in the latter being hospitalized with injuries. The patient had presented with learning difficulties since early age and was referred to a school for children with intellectual disabilities. The diagnosis of WS was made when she was 15-years old. She demonstrates an “elf-like” face, supravalvar aortic stenosis (evaluated in the past by cardiology) and growth retardation, which are present in up to 80% of individuals with WS [1]. She has also mental retardation, with learning deficits, social disinhibition and an unusual aptitude for music.

There is no evidence of prior psychiatric illness or symptoms. There is also no case of mental or neurologic disease in her family.

The psychotic symptoms improved gradually, and on day 7 at our unit they were no longer present. She was discharged 21 days after her admission to our unit.

## Discussion

In this presenting case, the clinical findings, namely the presence of persecutory delusions, auditory and verbal hallucinations, soliloquies, psychomotor agitation and an abrupt change of previous interpersonal functioning, suggest the diagnosis of psychosis.

According to the literature, psychosis is an uncommon comorbidity among patients with WS. Therefore, exhaustive medical investigation was required in order to exclude possible organic causes of psychosis, which initially seemed more plausible. However, as described before, none of the examinations that were conducted found any evidence of medical illness. At this point, it seems plausible to assume the diagnosis of nonorganic psychosis in this patient.

This comorbidity might represent independent pathophysiological processes between the two disorders. This seems more plausible because the association between these two is rare, according to the available literature. However, one cannot categorically exclude a pathological link between them.

There was no evidence of mood disorder at any time, either during the evaluation or in her past history, that could suggest the diagnosis of a psychosis. However, this patient has an intellectual disability that makes clinical evaluation of her symptoms hard to assess, which in turn complicates the differential diagnosis among several types of psychosis.

Another interesting aspect is the temporal relation between the arising of the psychotic symptoms and the reported events relating to family relations and routines. It is known that some events can act as important stressors in the development of psychotic symptoms, especially in acute psychotic episodes. In this case, we can only speculate about the role of those events in the development of psychosis. However, one could eventually link the accident in which she fractured her leg with the psychiatric symptoms. On this topic, we must remember that those symptoms were already present before that accident, and that she left home alone probably in the context of psychosis, but it is possible that the injury could have acted as an aggravator of those symptoms. Finally, it seems very unlikely that the psychotic symptoms were triggered or aggravated by analgesic therapy.

## Conclusion

The association between psychosis and WS is rare [5]. Here we describe a case of a woman for whom these two disorders seemed to coexist. Data in the literature on this topic are scarce and, therefore, this case report can contribute to the development of our knowledge on mental comorbidities in patients with WS.

## Consent

Written informed consent was obtained from the patient’s family for publication of this case report. A copy of the written consent is available for review by the Editor-in-Chief of this journal.

## Abbreviations

t.i.d.: Three times a day; WS: Williams syndrome.

## Competing interests

The authors declare that they have no competing interests.

## Authors’ contributions

Both authors were equally involved in the collection of data and in drafting the manuscript. Both authors read and approved the final manuscript.

## References

[B1] Meyer-Lindenberg A Mervis CB Berman KF Neural mechanisms in Williams syndrome: a unique window to genetic influences on cognition and behaviour Nat Rev Neurosci 2006 7 380 393 10.1038/nrn1906 16760918

[B2] Pober BR Wang E Caprio S Petersen KF Brandt C Stanley T Osborne LR Dzuria J Gulanski B High prevalence of diabetes and pre-diabetes in adults with Williams syndrome Am J Med Genet C Semin Med Genet 2010 154C 291 298 10.1002/ajmg.c.30261 20425788 PMC2882962

[B3] Huang L Sadler L O’Riordan MA Robin NH Delay in diagnosis of Williams syndrome ClinPediatr (Phila) 2002 41 257 261 10.1177/000992280204100410 12041723

[B4] Morris CA Williams syndrome http://www.ncbi.nlm.nih.gov/books/NBK1249/

[B5] Stinton C Tomlinson K Estes Z Examining reports of mental health in adults with Williams syndrome Res DevDisabil 2012 33 144 152 10.1016/j.ridd.2011.09.002 22093659

[B6] Joseph C Landru MM Bdeoui F Gogly B Dridi SM Periodontal conditions in Williams Beuren syndrome: a series of 8 cases Eur Arch Paediatr Dent 2008 9 142 147 10.1007/BF03262626 18793597

[B7] American Academy of Pediatrics Health care supervision for children with Williams syndrome Pediatrics 2001 107 1192 1204 11331709

[B8] Leyfer OT Woodruff-Borden J Klein-Tasman BP Fricke JS Mervis CB Prevalence of psychiatric disorders in 4 to 16-year-olds with Williams syndrome Am J Med Genet B Neuropsychiatr Genet 2006 141B 615 622 10.1002/ajmg.b.30344 16823805 PMC2561212

